# Tailoring desensitization in intestinal transplants: C1q guided protocol for highly sensitized recipients

**DOI:** 10.1016/j.intf.2026.100371

**Published:** 2026-05-19

**Authors:** Vighnesh Venkatasamy, Jennifer Garcia, Amay Banker, Mahmoud Morsi, Arinc Ozturk, Gennaro Selvaggi, Akin Tekin, Rafael Miyashiro, Hugo Kaneku, Phillip Ruiz, Rodrigo Vianna, Adela Mattiazzi

**Affiliations:** a Department of Surgery, Division of Transplant Surgery, University of Miami/Jackson Health System, Miami, FL, USA; b Department of Pediatrics, Division of Gastroenterology, Hepatology and Nutrition, University of Miami/Jackson Health System, Miami, FL, USA; c Department of Surgery / Pathology, University of Miami/Jackson Health System, Miami, FL, USA; d Department of Medicine, Division of Nephrology, University of Miami/Jackson Health System, Miami, FL, USA

**Keywords:** Intestinal transplantation, Desensitization, Modified multi-visceral transplantation, high sensitization

## Abstract

**Background:**

Sensitized candidates for isolated intestinal or modified multivisceral transplantation (MMVT) face prolonged wait times and higher rejection risk from donor-specific antibodies (DSA). While desensitization is established in kidney transplantation, data in intestine/MMVT are limited.

**Case report:**

Three adults with cPRA ≥ 95% underwent desensitization with Rituximab, plasma exchange, and monthly infusions of IgG, guided by C1q DSA testing. All three patients were transplanted—two with negative crossmatches and one despite a positive B-cell crossmatch given C1q-negative DSAs. No early AMR occurred; one developed mild acute cellular rejection, responsive to therapy. Infectious complications were common but manageable. Outcomes varied: one died nearly four years post-transplant from non-adherence leading to infections and severe rejection, one succumbed to necrotizing fasciitis at 10 months with a functional graft, and one remains alive with excellent graft function.

**Conclusion:**

C1q-guided desensitization enabled successful transplantation in highly sensitized isolated intestine/MMVT recipients, supporting its feasibility. Further clinical studies are warranted.

## Introduction

HLA sensitization remains a major barrier to successful isolated intestine and modified multivisceral transplantation. Candidates with elevated calculated panel reactive antibody (cPRA) levels face prolonged wait times and increased risk of rejection due to pre-formed donor-specific antibodies (DSA). Although multiple desensitization strategies have been developed in kidney transplantation, their application to isolated intestine or modified multivisceral transplantation is less well defined. [1] Moreover, the existing literature often shows inconsistencies in treatment approaches, with considerable variability across patients. [2], [3], [4], [5]

In this report, we describe our institutional experience using a C1q-guided desensitization framework in three highly sensitized isolated intestine or modified multivisceral transplant recipients (cPRA ≥95%). We focus on patient selection, immunologic risk stratification, donor acceptance decisions, and early post-transplant outcomes. This case series illustrates the application and evolution of a structured desensitization approach in a population with limited existing guidance. Institutional review board approval and informed patient consent was obtained.

## Institutional desensitization protocol

**Fig. 1:**
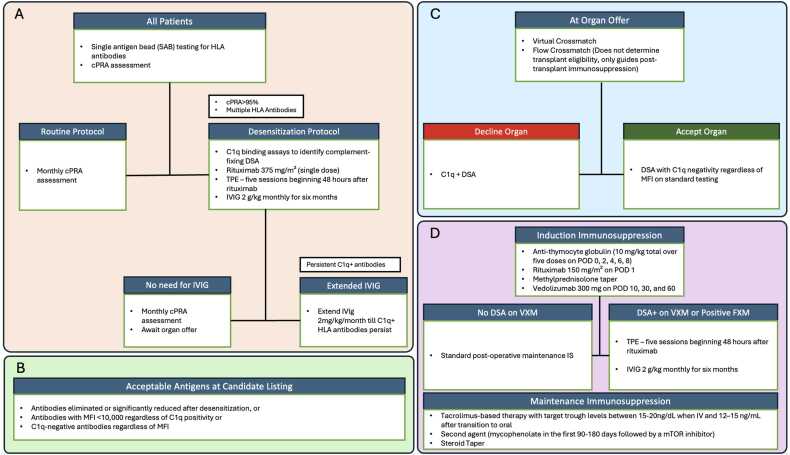
C1q-guided desensitization and immunologic decision framework for highly sensitized intestinal transplant candidates. Abbreviations: cPRA – Calculated Panel Reactive Antibody; DSA – Donor-Specific Antibody; FXM – Flow Cytometry Crossmatch; HLA – Human Leukocyte Antigen; IVIG – Intravenous Immunoglobulin; VXM – Virtual Cytometry Crossmatch; IS Immunosuppression; MFI – Mean Fluorescence Intensity; mTOR – Mammalian Target of Rapamycin; SAB – Single Antigen Bead; TPE – Therapeutic Plasma Exchange.

This institutional desensitization protocol represents an evolving framework refined over time rather than a rigid algorithm applied uniformly from inception (Fig. 1).

### Patient selection

Candidates were considered for desensitization if they had:

• cPRA ≥ 95%
• Persistent Class I and/or Class II HLA antibodies limiting donor availability
• No contraindications to intensified immunomodulatory therapy

### Immunologic assessment

All patients underwent:

• Single antigen bead (SAB) testing for HLA antibodies
• Serial cPRA assessment (monthly)
• Serial C1q binding assays to identify complement-fixing DSA

Antibodies were classified by mean fluorescent intensity (MFI) and C1q status. C1q positivity was used as a key marker of immunologic risk.

### C1q-guided desensitization strategy

The desensitization strategy consisted of:

• Rituximab 375 mg/m² (single dose)
• Therapeutic plasma exchange (TPE) – five sessions beginning 48 h after rituximab
• IVIG 2 g/kg monthly for six months (extended if C1q+ antibodies persisted)

Serial C1q testing was used to guide continuation or escalation of therapy.

### Donor acceptance criteria

• Acceptable donor antigens at time of candidate listing included:

• Antibodies eliminated or significantly reduced after desensitization, or
• Antibodies with MFI < 10,000 for C1q positive antigens or C1q-negativity regardless of MFI
• At organ offer, a Virtual crossmatch (VXM) was used to confirm compatibility:

• DSA that were C1q-negative were considered acceptable regardless of MFI on standard testing.
• A flow crossmatch (FXM) was performed on admission but was not used to determine transplant eligibility; results guided continuation or escalation of ISP therapy.

### Surgical considerations

• All isolated intestine and MMVT grafts included the colon till the splenic flexure.
• All surgeries were performed in two stages with temporary abdominal closure during the index transplant, followed by a planned re-exploration and closure 48 h later.
• Routine ileostomy was selectively performed in isolated intestinal recipients, one of the indications being highly sensitized recipients for ease of endoscopy and biopsy.

### Post-transplant immunosuppression and monitoring

Induction:

• Anti-thymocyte globulin (10 mg/kg total over five doses on post-operative days (POD) 0, 2, 4, 6 and 8)
• Rituximab 150 mg/m² on POD 1
• Methylprednisolone taper
• Vedolizumab 300 mg on POD 10, 30, and 60
  If DSA on VXM, or a positive FXM –
• Therapeutic plasma exchange (TPE) – five sessions beginning 48 h after rituximab
• IVIG 2 g/kg monthly for six months

Maintenance:

• Tacrolimus-based therapy targeting trough levels of 15–20 ng/mL during IV therapy and 12–15 ng/mL after transition to oral dosing during the first post-transplant month, with tapering thereafter
• Second agent: mycophenolate during the first 90–180 days followed by transition to an mTOR inhibitor
• Steroid taper by three months, when clinically feasible

Monitoring:

• Endoscopy with biopsy was performed for clinical indication (“for-cause”) rather than routine surveillance.
• DSA monitoring was performed weekly for the first 6 weeks, biweekly for the subsequent 6 weeks, and then monthly during the first post-transplant year.
• C4d staining was performed on all biopsy specimens.
• Positive DSA prompted IVIG (500 mg weekly for 4 weeks followed by monthly dosing for 3 months), with adjunct TPE or rituximab based on clinical findings

### Case series

#### Patient #1

Patient 1 was a 44-year-old female with intestinal failure (IF) due to severe gastroparesis requiring gastrostomy for decompression, jejunostomy feeding, and eventual total parenteral nutrition (TPN) dependence. Her course was complicated by refractory peptic ulcer disease, multiple CLABSIs, and sepsis. She was listed for a MMVT in 2017. At evaluation, she had a cPRA of 100% and C1q-fixing antibodies to B7, B81, and DR4. Over four years, several offers were initially declined due to positive VXM.

**Fig. 2:**
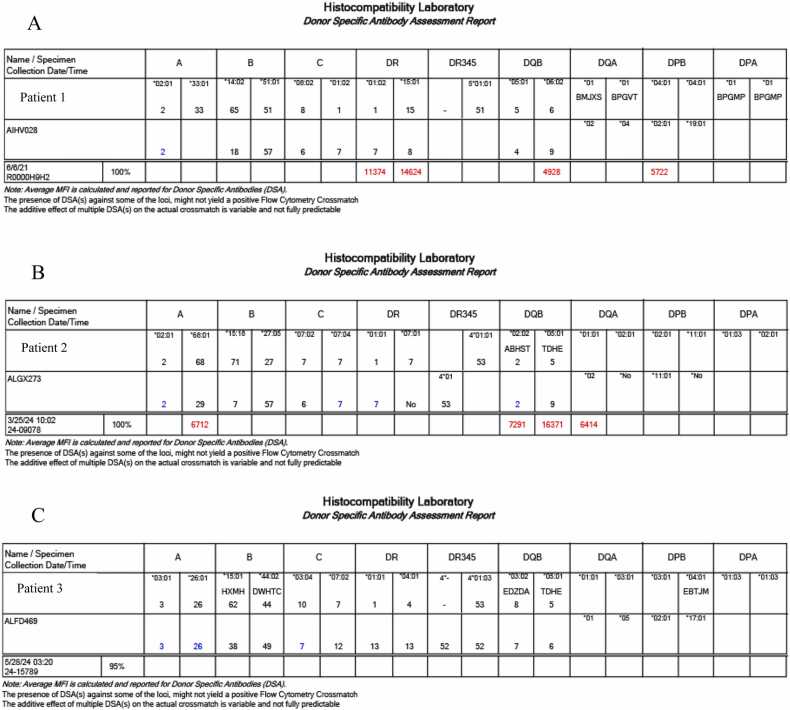
Complete virtual crossmatch results for all three cases.

In 2021, she began our desensitization protocol as described above. Serial monitoring of her cPRA and antibody levels revealed a downward trend following desensitization. In August 2021, she received an offer with the VXM demonstrating multiple DSAs (DR7, DR8, DQ, and DPB02:01) (Fig. 2A) but all were C1q negative. She underwent a MMVT as per the institutional protocol.

The FXM obtained on admission later resulted positive for B cells (6.48) and guided post-transplant immunosuppressive management, including five cycles of TPE and IVIG infusions; however, IVIG doses scheduled for months 3 and 4 were missed. Her postoperative course was complicated by a gastro-gastric leak and adenovirus infection treated with cidofovir. She was successfully weaned off TPN and discharged after two months with no evidence of rejection.

At four months, a ‘for cause’ endoscopy showed ileal necrosis attributed to adenovirus infection without rejection. Ileostomy reversal occurred at six months. At one-year post-transplant, she developed mild acute cellular rejection (ACR). Although antibody-mediated rejection (AMR) was not histologically confirmed, empiric therapy was initiated in the setting of rising DSA in this highly sensitized recipient. IVIG was administered initially; however, due to persistent DSAs, rituximab (375 mg/m²) was subsequently given. Over the following two years, she had recurrent infections and concerns for non-adherence with immunosuppression. Nearly four years post-transplant, she presented with severe ACR and multiorgan failure and died despite ATG therapy. No AMR was identified.

#### Patient #2

Patient 2 was a 48-year-old female with Crohn’s disease and IF following extensive bowel resections, complicated by recurrent CLABSIs. At evaluation for an isolated intestinal transplant, she was found to be highly sensitized (cPRA 100%) with peak Class I DSA (A1) of 31,180 MFI and Class II DSA (DQA1*05:05) of 26,721 MFI. Pre-treatment C1q assay showed multiple class I and II C1q-fixing antibodies. She received our institutional desensitization protocol mentioned above. Four months after the initial desensitization treatment, she underwent two additional cycles of TPE at the time of a potential organ offer. Monthly IVIG infusions were also extended beyond the initially planned six months due to persistently elevated DSA levels on serial C1q testing.

In July 2024, she received an organ offer with DSAs detectable on VXM (Fig. 2B), that were all C1q-negative and she underwent an uneventful isolated small bowel transplant. A FXM obtained on admission was negative, but given DSA on the VXM, induction ISP was per protocol with additional TPE and IVIG. Maintenance ISP consisted of tacrolimus, mycophenolic acid, and oral methylprednisolone with latter transitioned to budesonide given her Crohn’s disease.

Her post-operative course was uncomplicated. For cause endoscopies and biopsies revealed no rejection, and she was discharged two months post-transplant. She experienced a few readmissions for dehydration and AKI secondary to high ostomy output, without evidence of rejection. Ileostomy was successfully reversed at six months. One-year post-transplant, she remains rejection-free with a functional allograft. All post-transplant C1q-DSA tests have been negative to date.

#### Patient #3

Patient 3 is a 53-year-old female with history of colon cancer treated with chemotherapy, radiation and a low anterior resection. In 2011, she experienced a volvulus and small bowel strangulation, which necessitated a near total enterectomy resulting in IF. IF course was complicated by CLABSI with sepsis and by stroke in 2019.

She was highly sensitized (cPRA 95%) with peak Class I antibody (A23) of 29,000 MFI. C1q testing identified complement-binding anti-HLA antibodies to A2, A23, A24, A68, and A69. She underwent the same desensitization protocol mentioned above. Serial C1q testing showed persistent C1q+ antibodies, prompting an extension of IVIG therapy beyond the originally planned six months.

In June 2024, she received an acceptable organ offer without DSA’s and a negative VXM (Fig. 2C). She underwent an uneventful isolated small bowel transplant with a permanent end colostomy due to the inability to find a healthy stump of the native rectum. Given no DSAs and negative FXM obtained on admission, she did not receive any additional TPE, or IVIG infusions. Her maintenance IS was per protocol.

Her course was complicated by septic shock from infected intra-abdominal hematomas and urologic obstruction requiring interventions. She was discharged home three months post-transplant and for cause endoscopies confirmed her to be rejection-free at nine months. At 10 months post-transplant, she was transferred from an outside hospital with necrotizing fasciitis of the upper extremity and septic shock. She underwent multiple debridement’s, but her condition continued to decline, and her family opted for comfort care.

## Discussion

Sensitization significantly complicates organ allocation and outcomes for intestinal transplant recipients. In this report, we outline our center’s experience with desensitization in highly sensitized patients awaiting isolated intestine and MMVT.

### Immunology of intestinal transplants

The intestinal mucosa is in constant contact with foreign antigens from food and commensal microorganisms. Intestinal allografts must therefore tolerate benign antigens while simultaneously maintaining immunologic vigilance against pathogens. [6] This balance is regulated by the innate immune system through NOD2 proteins, and chemokines and cytokines signaling pathways that ensure a steady flow of regulatory T cells within the intestine. [7] Immediately after transplantation, there is an intense bidirectional trafficking of immune cells—donor lymphocytes migrate into the recipient’s circulation and vice versa, triggering both systemic and local immune responses. Immune cells within the graft maintain a balance between the effector and regulatory immune responses and only those allografts that achieve this balance can survive long-term. [8], [9]

Formation of DSA, particularly those that fix complement, increases the risk of rejection and graft loss. ^10^ The use of T-cell depleting induction immunosuppression regimens and long-term use of tacrolimus has been effective in preventing ACR. [10] However, AMR remains a major challenge in intestinal transplantation. [11] Identifying pre-formed DSAs and monitoring for the development of de-novo DSAs allows for timely implementation of antibody reducing strategies to minimize the risk of graft loss.

### Pre-transplant testing: rationale of the C1q assay

Our desensitization protocol begins with identifying complement-binding antibodies using the C1q assay. Traditional HLA antibody testing detects all subclasses of IgG regardless of their ability to bind complement. ^1^ Evidence suggests that only complement binding subclasses of IgG (IgG1 and IgG3) are likely to cause allograft injury. [12] The C1q assay, identifies complement-fixing antibodies by adding exogenous C1q, which binds to the Fc region of these IgG antibodies. This binding is then detected using a fluorescent-conjugated anti-human C1q antibody.

The clinical utility of the C1q assay has primarily been tested in kidney transplants. One study found that C1q+ DSA, combined with high IgG levels (MFI > 5000), significantly increased the likelihood of a positive FXM. Moreover, C1q+ DSA were shown to be highly specific (95.8%) for rejection risk. [13] In an analysis of 1016 sensitized kidney transplant recipients, Loupy et al. reported higher rates of AMR and significantly higher incidence of graft loss in recipients with C1q+ DSA compared to those with C1q-negative DSAs or no DSAs. [14] Another study indicated that C1q-fixing antibodies were more predictive of AMR than high IgG levels alone. [15] Emerging data also suggests that the C1q assay can predict poor outcomes in other organ transplants, such as liver and pancreas. [16], [17] Given these findings, we have adapted the C1q assay to identify and monitor anti-HLA antibodies in our isolated intestinal and MMVT recipients.

### Antibody reducing strategies

Our antibody-reducing strategy involved a combination of Rituximab, TPE, and IVIG. The treatment began the therapy with a single dose of Rituximab (375 mg/m^2^) followed by five cycles TPE initiated 48 h later. Monthly IVIG infusions at 2 g/kg/dose were administrated to all patients for at least 6 months. Rituximab, a monoclonal antibody targeting CD20 + B cells, was used to reduce the number of B cells. TPE was used to physically remove circulating antibodies, while IVIG further modulated the immune system. [18] In our case series, there were no AMR at one year, and there was only one episode of mild ACR which was successfully treated.

There are several desensitization protocols used in transplantation tailored to the patients’ degree of sensitization. [19], [20], [21] Some protocols use high-dose IVIG therapy alone or in combination with TPE to reduce HLA antibodies. Other protocols include the use of Bortezomib, a proteasome inhibitor that targets plasma cells, to reduce antibody production. [22] Eculizumab, a monoclonal antibody targeting the terminal components of the complement system, has also been utilized to prevent rejection. [23] More recently, C1q esterase inhibitors such as Berinert have been explored as an antibody-reducing strategy. [24] While these therapies are promising, there is limited data on their effectiveness in intestinal transplant candidates.

In 2006, Gondolesi et al. first introduced a stepwise desensitization protocol for isolated intestinal transplant candidates. They described the use of three escalating doses of IVIG, followed by four doses of Rituximab for patients with a persistently elevated PRA. In their experience, 3 out of the 6 patients who underwent desensitization were successfully transplanted. [3] Another group described two living donor intestinal transplant candidates who successfully underwent desensitization using 3 and 7 sessions of TPE, followed by low-dose IVIG administration. [25] Recently, a team in Mount Sinai, NY outlined a risk-stratified, stepwise desensitization protocol for isolated intestinal transplants. Their protocol included two doses of Rituximab and IVIG for all sensitized patients, with TPE and Bortezomib added for those with cPRA > 60% after initial treatment. Their results showed a reduction in the mean PRA from 62.1% to 48.8%. Seven of eight patients were successfully transplanted, with a 71.4% graft survival rate at 2.5 years. [2] Importantly, none of these protocols incorporated C1q testing to risk-stratify DSAs, highlighting the novelty of our C1q-guided desensitization strategy.

### End point of desensitization strategies

There is currently no universally accepted endpoint for desensitization in highly sensitized intestinal transplant candidates. In our approach, we relied on trends in cPRA and serial C1q assays to evaluate antibody burden. When preformed DSAs remained elevated, monthly IVIG infusions were extended. Acceptable donor antigens were defined as those with an initial MFI < 10,000 or C1q negativity. At the time of organ offer, a VXM was used to screen for preformed DSA, and DSAs that were C1q-negative regardless of MFI on standard testing were considered acceptable. A FXM was obtained on admission but did not determine transplant eligibility; instead, results were used to guide post-transplant immunosuppressive management. In our series, two of three patients required additional post-transplant TPE based on VXM or FXM findings.

There is evidence supporting the use of allografts with a positive FXM in intestinal transplantation. Kubal et al. demonstrated no difference in outcomes for FXM positive transplant recipients. Similar to ours, their protocol included one dose of Rituximab (150 mg/m^2^) in all patients. In their series, cross match positive patients received five cycles of TPE, a higher dose or subsequent dose of Rituximab, and IVIG infusions. [26]

### Post-transplant immunosuppression

Our post-transplant immunosuppression protocol for liver-free intestinal grafts includes induction with ATG (10 mg/kg divided in five equal doses), Rituximab (single dose 150 mg/m²), methylprednisolone taper over 14 days, and Vedolizumab (300 mg on POD 10, 30 and 60). Methylprednisolone is transitioned to oral steroids and is tapered by 3 months. Maintenance immunosuppression consists of tacrolimus and a second agent. Tacrolimus is started as a continuous IV infusion with trough goals of 15–20 ng/mL and transitioned to oral with trough goals of 12–15 ng/mL in the first month. Trough goals are progressively decreased over the first year. The second agent is mycophenolate mofetil for the first six months (to facilitate wound healing) and is later replaced by an mTOR inhibitor (either everolimus or sirolimus) allowing lower tacrolimus trough levels without increasing rejection risk.

Vedolizumab is a humanized monoclonal antibody targeting the α4β7 integrin preventing the migration of activated T cells into the gut by blocking interaction with Mucosal Addressin Cell Adhesion Molecule-1 (MadCAM-1). [27] It is a gut specific immunomodulator with little systemic effects making it an attractive adjunct in intestinal transplantation. The dynamics of α4β7^+^ cells in the graft suggest that autoreactive leukocytes, which may initiate a rejection episode, could be inhibited or downregulated during induction. Additionally, Vedolizumab targets FoxP3^+^ regulatory T cells (Treg), implying that α4β7^+^ cells may play a role in ACR. [28] Recent evidence suggests that its mechanism of action may also include modulation of components of the innate immune system. It is now recognized that plasmablasts, which typically leave the germinal center and differentiate into antibody secreting plasma cells and memory B cells, also express high levels of α4β7. By affecting the gut trafficking of these cells, Vedolizumab can potentially dampen not only the production of antibodies by plasma cells but also the availability of memory cells that produce antibody when exposed to the pathogen (donor) again. [29] In our single center experience, no patients receiving Vedolizumab as part of our induction protocol since 2018 have had biopsy proven AMR.

### Post-transplant monitoring

We monitor for persistent pre-formed and de-novo DSA in all transplant recipients with decreasing frequency throughout the post-transplant course with at least monthly surveillance during the first year. In those with positive DSA, addition C1q-fixing antibodies are obtained. Positive DSA, regardless of MFI values, prompt us to initiate interventions initially with IVIG (2 g/kg/month). Adjunct Rituximab and/or TPE are utilized depending on clinical relevance.

The clinical application of the C1q assay was reported in kidney transplantation by Loupy et al., who demonstrated that patients with complement-binding DSA after transplant had the lowest 5-year graft survival rate (54%) compared to patients with non-complement-binding DSA (93%) and those without DSA (94%). [14] Similarly, Schaefer et al. studied 80 pre-sensitized kidney recipients (cPRA ≥85%) who underwent desensitization using plasmapheresis and anti-CD20 therapy. Post-transplant, patients with C1q+ DSA had significantly higher rates of AMR and graft loss due to AMR compared to those with C1q− or no DSA (86%vs. 33% vs. 0% for AMR) and (86% vs. 0% vs. 0% for graft loss), respectively. [30]

In conclusion, HLA sensitization continues to present significant barriers to successful isolated intestine and MMVT transplantation. The immunological complexity of the intestinal graft, combined with the high prevalence of sensitized candidates, necessitates an individualized approach. Our protocol, which incorporates C1q-based risk stratification, a combination of antibody-lowering therapies (Rituximab, TPE, IVIG), and a carefully constructed immunosuppressive regimen with Vedolizumab, has shown encouraging results in preventing AMR. Continued refinement of desensitization strategies and long-term monitoring practices will be critical for optimizing outcomes in this vulnerable patient population.

## Ethical clearance

Institutional review board approval has been obtained with their letter number 20140129 dated 5/19/2016

## Funding

This research did not receive any specific grant from funding agencies in the public, commercial or not-for-profit sectors.

## CRediT authorship contribution statement

**Phillip Ruiz:** Visualization, Supervision, Investigation, Conceptualization. **Hugo Kaneku:** Writing – review & editing, Visualization, Methodology, Conceptualization. **Rafael Miyashiro:** Writing – review & editing, Supervision, Data curation, Conceptualization. **Akin Tekin:** Writing – review & editing, Supervision, Data curation, Conceptualization. **Gennaro Selvaggi:** Writing – review & editing, Resources, Conceptualization. **Arinc Ozturk:** Project administration, Methodology, Data curation, Conceptualization. **Mahmoud Morsi:** Writing – review & editing, Project administration, Conceptualization. **Amay Banker:** Writing – review & editing, Writing – original draft, Methodology, Data curation, Conceptualization. **Jennifer Garcia:** Writing – review & editing, Resources, Project administration, Conceptualization. **Mattiazzi Adella:** Writing – review & editing, Project administration, Methodology, Investigation, Data curation, Conceptualization. **Vighnesh Venkatasamy:** Writing – review & editing, Writing – original draft, Methodology, Formal analysis, Conceptualization. **Rodrigo Vianna:** Writing – review & editing, Supervision, Project administration, Conceptualization.

## Declaration of Competing Interest

The authors declare that they have no known competing financial interests or personal relationships that could have appeared to influence the work reported in this paper.
